# The effect of progesteron for expression delta (δ) opioid receptor spinal cord through peripheral nerve injury

**DOI:** 10.1016/j.amsu.2022.103376

**Published:** 2022-02-11

**Authors:** Bambang Priyanto, Rohadi Muhammad Rosyidi, Andi Asadul Islam, Agus Turchan, Yusra Pintaningrum

**Affiliations:** a Department of Neurosurgery Medical Faculty of Mataram University, West Nusa Tenggara General Hospital, Mataram, Indonesia; b Doctorate Program, Faculty of Medicine, Hasanuddin University, Makassar, Indonesia; c Department of Neurosurgery, Faculty of Medicine, Hasanuddin University, Makassar, Indonesia; d Department of Neurosurgery, Dr. Soetomo General Hospital Medical Center, Faculty of Medicine, Airlangga University, Surabaya, East Java, Indonesia; e Department of Cardiology and Vascular Medicine, Medical Faculty of Mataram University, West Nusa Tenggara General Hospital, Mataram, Indonesia

**Keywords:** Peripheral nervous system, Pain, Progesterone, Delta (δ) opioid receptors

## Abstract

**Background:**

Neuropathic pain is a major problem to date because of its high prevalence and lack of effective treatment. Neuropathic pain processes can be influenced by many factors and at various levels of the nervous system, including progesterone and the opioid system. The various mechanisms of the effect of progesterone on pain are still controversial, while the effect of progesterone on the activation of the opioid system also needs to be proven. This study aimed to determine the effect of progesterone on pain through the modulation mechanism of the opioid system.

**Methods:**

This research is a completely randomized experimental study using male wistar rats aged around three months at the Experimental Animal Laboratory, Department of Medical Biochemistry, Faculty of Medicine, Airlangga University.

**Results:**

The result was analyzed by using statistical analysis of two independent samples (*t*-test). The t value was obtained at 6.880, p = 0.000 (p < 0.05).

**Conclusion:**

It was shown that there was a significant difference in the delta (δ) opioid receptor expression between the control group and the progesterone group, which indicated that progesterone causes an increase in the delta (δ) opioid receptor expression in the spinal cord.

## Highlights

• Experimental study on mice subjects to determine the difference delta (δ) opioid receptors in the spinal cord.
• Determine the effect of progesterone on pain through the modulation mechanism of the opioid system.
• The administration of progesterone has a positive effect on the expression of delta (δ) opioid receptors.

## 1 Introduction

The incidence of peripheral nerve injury in the United States annually ranges from 200,000 to 400,000 people [1,2]. In the European Union, there are about 300,000 spinal cord injuries with 11,000 new cases each year. Epidemiological studies suggest that about two-thirds of spinal cord injury cases suffer from chronic pain and one-third of these suffer from severe chronic pain [3].

Nerve injury or dysfunction in the central and peripheral nervous systems is a major cause of neuropathy such as *allodynia* and hyperalgesia. This abnormal pain sensation is associated with various complex physiological changes in the central and peripheral nervous systems. Physiological changes include *spontaneous neuron discharging*, changes in ion gate expression, *sprouting* primary afferent neuron, peripheral and central sensitization, spinal reorganization and changes in descending pain inhibitory pathways [4].

Many studies suggest an important influence of sex differences on pain perception. This difference is seen from neonates to adults. The difference in pain perception between men and women is thought to be due to differences in response to opioids and differences in hormonal levels [7]. Peripheral sex steroids have been shown to affect central opioid activity, in addition, changes in steroid levels during pregnancy can modulate the opioid system. The dynorphine/kappa opioid system has been shown to increase the nociceptive threshold during pregnancy [8]. Drugs that are known to be effective for nociceptive pain and inflammatory pain are not all effective for relieving neuropathic pain. Even drugs belonging to the morphine class, which are often used for nociceptive pain, are also less effective for neuropathic pain [6].

In general, opioid agonists are more potent in one sex than in the other. Animal clinical studies have shown that -opioid agonists are more potent in males than females and that testosterone administration increases sensitivity to mu and kappa antinociceptives, whereas the modulating effects of estrogen and progesterone depend on the type of opioid agonist administered [7]. Clinical studies in humans to mention that sensitivity to pain in women already puberty increased especially in the menstrual cycle luteal phase (progesterone levels are high) compared to the follicular phase (progesterone levels are low) [9].

The spinal cord is the center for the formation of various neurosteroids such as *pregnenolone, dehydroepiandrosterone, progesterone* and *allopregnenolone* [[10], [11], [12]]. Progesterone receptors in the spinal cord are thought to have a very important role in the management of neuropathic pain. Increased levels of progesterone during pregnancy can trigger activation of the spinal opioid system, increase the release of endogenous opioids and decrease sensitivity to pain [8]. Other researchers have shown otherwise that progesterone receptor antagonists have the potential to inhibit pain in patients with neuropathic pain [6].

Changes in sensitivity to pain as a result of progesterone administration is still controversial. Frye proved a shortening of the latency time of pain after administration of progesterone, while Gordon proved otherwise. The affinity of kappa opioid receptors decreases after administration of progesterone, so that the decrease in pain sensitivity cannot be explained by the mechanism of the effect of progesterone on opioid receptors. Another mechanism proposed by Frye is that progesterone metabolites can increase pain sensitivity through interaction with GABA [8]. From the description above, it can be concluded that This study aimed to determine the effect of progesterone on pain through the modulation mechanism of the opioid system. It is hoped that knowing the relationship between the two can be an alternative for pain management after peripheral nerve injury.

## 2 Materials and method

This type of study is an experimental study with a completely randomized design. The study was conducted at the Animal Laboratory, Department of Medical Biochemistry, Faculty of Medicine, Airlangga University. This study aimed to determine the effect of progesterone administration on the expression of spinal cord delta (δ) opioid receptors. Opioid receptor expression was determined by immunohistochemical examination by counting the number of neuron cells that gave positive expression in every 100 neuron cells with clear nuclei.

### 2.1 Animal unit

Unit The experimental unit was male wistar rats aged ±3 months in the Animal Laboratory, Department of Medical Biochemistry, Faculty of Medicine, Airlangga University. Replication of each group was 6. These 16 experimental units in the form of male wistar rats aged approximately three months with initial body weight ranging from 152 to 190 g, the drop out rate was 25% (4 individuals). The left sciatic nerve was exposed by bluntly splitting the biceps femoris muscle under aseptic procedures and anesthesia with ketamine (40 mg/kg intraperitoneally). Using chromic cat gut 3.0 thread, the nerve was isolated from the surrounding tissue and lightly tied (the sciatic nerve was tied with a needle and the needle was removed) (reg. A.G.127.2 produced by ethicon). Fastening is done in four spots, each separated by 1 mm. Silk 3.0 was used to stitch the muscle and skin layers (reg O-9294, produced by B-Braun). To avoid infection, the wound was treated with an antiseptic solution and an antibiotic injection of ampicillin.

### 2.2 Correction factor

In anticipation of the experimental unit missing (drop out), a correction factor of 20% is used so that the number of replications per group becomes 7.55–8. So, the total replication is 12 mice. Then the mice were put into a complete randomized treatment group.

### 2.3 Drug treatment

The research subjects were randomly divided into two groups: progesterone group and control group. The progesterone using a 3 cc suspension of depoprogestin containing Medroxy Progesterone Acetate 150 mg/vial, produced by Harsen, Jakarta Indonesia. DKL Register Number 8307903243A1. Mice pain response is indicated by the reaction of mice in the form of squeaking, licking legs, struggling, or pulling the foot opposite the examined foot (contralateral). Expression of one of the opioid receptor subunits, which are distributed on the surface of the dorsal horn spinal cord; when there is neuronal damage to neuropathic pain. The preparations were taken from the lumbar spinal cord, and immunohistochemistry was examined, and the amount of opioid receptor expression was measured in the preparations. For the control group, gender, age, body weight, method of maintenance, neuropathy models, drug dosage, sampling techniques, and sample examination were analyzed.

### 2.4 Number of replication

The number of replications per group was six mice with two treatment groups so that the experimental unit needed was 12 animals. Replication per group was obtained using the Federer formula (1955) and a correction factor of 10% using the Higgins and Klimbaum formulas. Determination of the correction factor of 10%, based on pre-liminary research because at the end of the preliminary study, no experimental animals were found to drop out or die, and in this study also no experimental animals were found to drop out or die.

### 2.5 Hematoxylin-eosin (HE) staining

On the two weeks after treatment, the rat was euthanasia and surgically obtain spinal cord tissue, and then a histopathological preparation was made with HE staining and IHC preparation with the delta (δ) opioid receptors. HE preparations were observed with a 100x magnification light microscope to see the histopathology of the spinal cord and 200x magnification to identify neurons and to ensure the feasibility of these preparations for analysis by the IHC technique.

IHC microscope slide was observed with a 200 × magnification light microscope to identify neuron cells that can give a positive reaction to delta (δ) opioid receptors antibodies. Positive cells, if cytoplasm contains granules and cells are negative if cytoplasm contains without granules and clear.

### 2.6 Statistical analysis

The collected data were analyzed by one-way analysis of variance (ANOVA) tests to determine the potential of progesterone drug with a control group (+). Data is considered normal and homogeneous distribution if the normality and homogeneity test shows p > 0.05, then p > 0.05 data are analyzed by ANOVA. If the data is found to have a value of p < 0.05, the data are considered to be not normally distributed and not homogeneous. The Shapiro-Wilk test data (normality) p < 0.05 was tested with Brown Forsythe. The Levene test data (homogeneity) p < 0.05 was tested by Kruskal Wallis. ANOVA analysis results are said to be meaningful if the value of p < 0.05 is obtained. If the ANOVA analysis results are signif-icant, proceed with the Post Hoc LSD test. Statistical results were performed using SPSS 15.0.

## 3 Result

In this study, male wistar rats aged 3 months with initial body weight between 152 and 190 g were used. 8 animals were used randomly in each group, 4 of them dropped out due to infection and death. The complete characteristics of the experimental unit are shown in Table 1.

**Table 1:** Characteristic of sample.

No	Group	Body Weight (gram)			Result
		**Day 3 (pre- ligation)**	**Day 17 (pre- injection)**	**Day 31 (post- injection)**	
1	Progesteron	156	170	184	Mean body weight Day 3 : 172, 50 ± 14,896 Mean body weight Day 17 : 193, 33 ± 16,145 Mean body weight Day 31 : 213, 17 ± 23,008
2	Progesteron	171	179	184	
3	Progesteron	190	210	235	
4	Progesteron	187	192	222	
5	Progesteron	176	208	229	
6	Progesteron	155	201	225	
7	Control	155	184	210	Mean body weight Day 3 : 169, 17 ± 8612 Mean body weight Day 17 : 191, 83 ± 9065 Mean body weight Day 31 : 215, 67 ± 6976
8	Control	180	193	205	
9	Control	166	184	222	
10	Control	173	205	221	
11	Control	167	185	215	
12	Control	174	200	221	

The control group showed a mean positive expression of 25.83 ± 7.705% with n = 6, while in the progesterone group the average positive expression was 71.00 ± 14.114% with n = 6. The complete results of calculating the expression of -opioid receptors are shown in Table 2.

**Table 2:** Receptor delta (δ) opioid expression.

No	Positive	Percentage	Mean	SD
P1	67	67,00%	71,00%	14,114%
P3	51	51,00%		
P5	72	72,00%		
P6	76	76,00%		
P7	94	94,00%		
P8	66	66,00%		
K1	25	25,00%	25,83%	7705%
K3	17	17,00%		
K4	20	20,00%		
K6	33	33,00%		
K7	37	37,00%		
K8	23	23,00%		

**Fig. 1:**
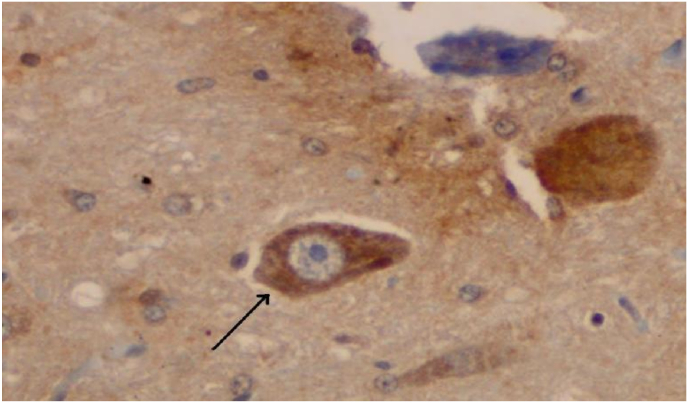
Neuron cells with positive expression of delta (δ) opioid Receptors (black arrow).

**Fig. 2:**
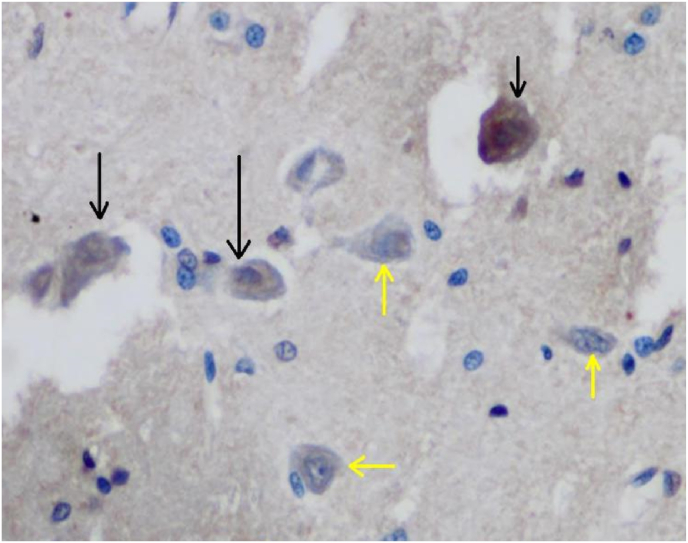
Neuron cell with positive expression of (delta (δ) opioid receptors (black arrow) and negative expression (yellow arrow). (For interpretation of the references to colour in this figure legend, the reader is referred to the Web version of this article.)

By using statistical analysis of two independent samples (*t*-test) the t value was obtained at 6.880, p = 0.000 (p < 0.05). So there was a significant difference in the expression of -opioid receptors between the control group and the progesterone group. It can be concluded that the administration of progesterone has a positive effect on the expression of delta (δ)-opioid receptors (Fig. 1 and Fig. 2).

## 4 Discussion

The mean initial body weight in the control group was 169.17 ± 8.612 g, while the progesterone group was 172.50 ± 14.896 g. There was an increase in body weight of experimental animals, namely the average in the control group increased to 191.83 ± 9.065 g, while the progesterone group increased to 193.33 ± 16.145 g beforeperformed chronic constriction injury was. At the end of the study, the average body weight in the control group increased to 215.67 ± 6.976 g, while in the progesterone group it increased to 213.17 ± 23.008 g.

The results of the normality test using the Kolmogorov-Smirnov test showed that the distribution of body weight at the three times mentioned above was normal. (control group p1 = 0.982; p2 = 0.756 and p3 = 0.744; the progesterone group showed p1 = 0.971; p2 = 0.988 and p3 = 0.586).

Based on the analysis of the effect of progesterone on the expression of delta (δ)-opioid receptors, the t-count value was 6.880, p = 0.000 (p < 0.05). So there was a significant difference in the expression of delta (δ)-opioid receptors between the control group and the progesterone group. It can be concluded that the administration of progesterone has a positive effect on the expression of delta (δ) opioid receptors.

Spinal cord injury results in disturbances in the transmission and integrity of sensory system information. Sensory system abnormalities will activate the endogenous opioid system which results in increased inhibition regulation and suppresses the onset of symptoms allodynia. On the other hand, increased activity of the opioid system will be inhibited by CCK activation, resulting in allodynia chronic [13].

After cutting the peripheral nerves, there was a marked increase in the activity of CCK and its receptors in the Dorsal Root Ganglion (DRG). This increase is due to increased synthesis and release of CCK from primary afferent fibers. This condition results in antagonistic effects of endogenous and exogenous opioids [13].

Chronic lesions of peripheral nerves cause a decrease in the analgesic potency of morphine which is thought to be due to decreased signal transduction activity atopioid receptors post-synaptic [10,11], increased morphine tolerance, decreased number of opioid receptors and activation of an antiopioid peptide cholecystokinin (CCK) [13].

Other studies suggest that post-peripheral nerve neuropathic pain occurs due to reduced sodium flow at the slow-type sodium gate, decreased refractory period of afferent axons, and reduced potassium flow resulting in hyperexcitability interfere with the patient's activities. This happens because there is an increase in chemical and mechanical sensitivity, cross-generation and *ectopic impulse firing.* [5] Neuropathic pain is chronic pain associated with various events such as nerve damage and mechanical suppression of nerve cells. Neuropathic pain is also found in several diseases that affect peripheral nerve function such as diabetes and malignancy [6].

Another study showed that there was an anatomical reorganization of the spinal cord in the form of cross-stimulation between type A fibers and type C fibers, termination of large myelinated Aβ nerve endings becoming more superficial in lamina II [14].

Studies in mice have shown that peripheral axon injury reduces the effect of mu opioid receptors and GABAergic inhibition on the spinal cord [10,11]. Immunohistochemical examination showed that after excision of peripheral nerves (axotomy) there was a decrease in the expression of mu and delta receptors on the soma of sensory neurons and their projection ends in the dorsal root ganglion [15].

Progesterone is included in the class of neurosteroids, where neurosteroid-specific functions are carried out by modulating the GABA system, NMDA and delta receptors. Progesterone is useful for regulating myelination, neuroprotection and growth of axons and dendrites [10,11]. The *anti*-hyperalgesia effect of progesterone is thought to occur at the spinal cord level where NMDA receptor suppression occurs, even though it is known that NMDA receptor activation is involved in persistent pain and hyperalgesia [16].

Peripheral nerve injury causes changes in neurosteroidogenesis in bothcomponents genomic and biochemical. Studies in rats have shown that neuropathic conditions lead to increased levels of theneurosteroid pregnenolone endogenousin the nervous system while plasma levels are unchanged. There is an increase in gene expression, redistribution and changes in the bioactivity of the P450scc enzyme, one of the enzymes that play a role in the biosynthesis of progesterone, during neurogenic pain strongly suggesting an endogenous mechanism that facilitates the body's adaptation to pain [10,11].

In pain, high levels of progesterone are strong stimulators of GABA receptors A. This is a crucial factor in the regulation of pain sensation and is an adaptive mechanism to reduce sensitivity to painful stimuli in neuropathic conditions [11,12].

The mechanism by which sex steroid hormones activate spinal opioid antinociceptive pathways is still unknown. Administration of estradiol and progesterone increased theopiate receptor binding densityby 52% andin the -endorphins preoptic area. The modulation of signal transduction through opioid receptors by estrogen and progesterone should also not be overlooked [17].

A delta of opioid receptors is seen on lamina I and II of the dorsal horn of the spinal cord as well as in the gray area around the central canal [17]. Spinal delta opioid receptor activity is required to increase pain threshold during pregnancy and hormonal simulation (hormone-stimulated pregnancy) [17]. In neuropathic pain, there is a decrease in the number of opioid receptors in areas that play a role in pain transmission, including the spinal cord [4].

Based on the analysis of the effect of progesterone on the expression of delta (δ)-opioid receptors, the t-count value was 6.880, p = 0.000 (p < 0.05). So there was a significant difference in the expression of -opioid receptors between the control group and the progesterone group. It can be concluded that the administration of progesterone has a positive effect on the expression of delta (δ)-opioid receptors.

These results indicate that progesterone causes an increase in the expression of delta (δ)-opioid receptors in the spinal cord, in accordance with previous studies which suggested an increase in the binding density of delta opioid receptors [17] and the expression of receptors delta (δ)-opioids in the arcuate preoptic area and nucleus after administration of progesterone and estrogen [18].

## 5 Limitation

This study had a drop out rate of 25% due to infection and death. Another possible cause is the study period which lasted for 31 days. It is necessary to add clinical and functional examinations that indicate the emergence of a neuropathic state, as well as monitoring of progesterone levels in the experimental animals.

Examination of the expression of delta (δ)-opioid receptors in this study used immunohistochemical techniques by manually counting the number of neurons that gave positive expressions. This has a subjective tendency because it depends on the experience of the pathologist. Another technique that might be used is to enter the pathology image in the form of digital photos which are then read in a computer program so as to reduce subjectivity, using immunofluorescence ortechniques in situ hybridization.

This study has not been able to explain how the mechanism of increasing the expression of opioid receptors after administration of progesterone. Nor can it explain whether increased expression of opioid receptors is associated with decreased neuropathic pain. For this reason, further research is needed using more complete variables and other techniques, for example by examining nociceptive behavior tests. The limitation of this study is that the research was carried out on experimental animals so that it needs to be continued in the future with clinical research involving others biomarkers for neuropathic pain. The preparation of this manuscript is in accordance with the ARRIVE Guideline [19].

## 6 Conclusion

There is an increase in the expression of opioid receptors on spinal cord posterior horn neurons after progesterone administration in peripheral neuropathic lesions. Further research is needed to determine the relationship between increased expression of opioid receptors and decreased neuropathic pain and mechanism of the increase in opioid receptor expression after administration of progesterone.

## Author contribution

BAM, RHA, AGT, and AAI wrote the manuscript and participated in the study design. BAM, RHA, AGT, AAI and YP drafted and revised the manuscript. BAM, RHA, AGT and AAI performed head trauma treatment and surgery. BAM, RHA, and YP performed bioinformatics analyses and revised the manuscript. All authors read and approved the final manuscript.

## 7 Registration of research studies

None.

## Guarantor

Rohadi Muhammad Rosyidi.

## Funding

No funding or sponsorship.

## Ethical approval

All procedure for Animal experiment has been approved by Animal Care and Use Committee (ACUC) Faculty of Veterinary Medicine, Airlangga University, Number: 077-KE.

## Consent

This manuscript does not involve human participants, human data, or human tissue.

## Declaration of competing interest

The authors declare that they have no conflict of interests.

## Provenance and peer review

Not commissioned, externally peer reviewed.

## Ethical approval

All procedure for Animal experiment has been approved by Animal Care and Use Committee (ACUC) Faculty of Veterinary Medicine, Airlangga University, Number: 077-KE.

## Sources of funding

No funding or sponsorship.

## Author contribution

BAM, RHA, AGT, and AAI wrote the manuscript and participated in the study design. BAM, RHA, AGT, AAI and YP drafted and revised the manuscript. BAM, RHA, AGT and AAI performed head trauma treatment and surgery. BAM, RHA, and YP performed bioinformatics analyses and revised the manuscript. All authors read and approved the final manuscript.

## Registration of research studies

Name of the registry:

Unique Identifying number or registration ID:

Hyperlink to your specific registration (must be publicly accessible and will be checked): None.

## Guarantor

Rohadi Muhammad Rosyidi.

## Consent

This manuscript does not involve human participants, human data, or human tissue.

## Declaration of competing interest

The authors declare that they have no conflict of interests.
